# The Complex Role of Autophagy in Melanoma Evolution: New Perspectives From Mouse Models

**DOI:** 10.3389/fonc.2019.01506

**Published:** 2020-01-10

**Authors:** Luca Di Leo, Valérie Bodemeyer, Daniela De Zio

**Affiliations:** Cell Stress and Survival Unit, Center for Autophagy, Recycling and Disease (CARD), Danish Cancer Society Research Center, Copenhagen, Denmark

**Keywords:** melanoma, autophagy, metabolism, GEMM, syngeneic mouse model

## Abstract

Despite tremendous efforts in the last decade to improve treatments, melanoma still represents a major therapeutic challenge and overall survival of patients remains poor. Therefore, identifying new targets to counteract melanoma is needed. In this scenario, autophagy, the “self-eating” process of the cell, has recently arisen as new potential candidate in melanoma. Alongside its role as a recycling mechanism for dysfunctional and damaged cell components, autophagy also clearly sits at a crossroad with metabolism, thereby orchestrating cell proliferation, bioenergetics and metabolic rewiring, all hallmarks of cancer cells. In this regard, autophagy, both in tumor and host, has been flagged as an essential player in melanomagenesis and progression. To pave the way to a better understanding of such a complex interplay, the use of genetically engineered mouse models (GEMMs), as well as syngeneic mouse models, has been undoubtedly crucial. Herein, we will explore the latest discoveries in the field, with particular focus on the potential of these models in unraveling the contribution of autophagy in melanoma, along with the therapeutic advantages that may arise.

## Introduction

Melanoma arises from the malignant transformation of melanocytes, the melanin-producing cells mainly found in the skin's epidermis (1). Due to its mutational burden and heterogeneity, melanoma is a particularly aggressive cancer and still remains a clinical challenge (2, 3). In recent years however, a better understanding of melanoma biology and the identification of key genetic alterations causing imbalances in cell proliferation signaling (e.g., *BRAF, NRAS, PTEN, CDKN2A*) have revealed novel targets and therapeutic strategies for this disease (1–4). In particular, this knowledge has led to the development of the BRAF/MEK inhibitors currently used for subsets of patients harboring specific mutations and immunotherapies that aim at reactivating immune T cells through the use of immune checkpoint inhibitors (antibodies against CTLA-4 and PD-1/PD-L1) (1, 3, 5).

**Figure 1:**
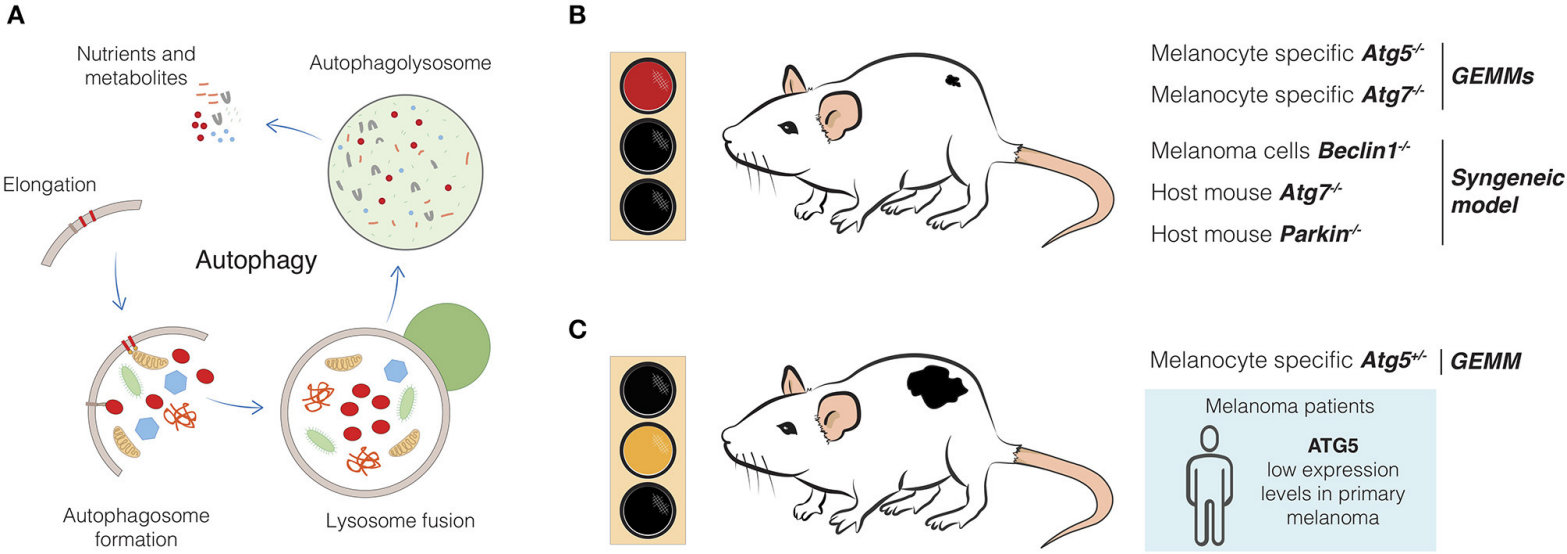
Effects of autophagy genes alteration in melanoma development. **(A)** Autophagy is a complex, dynamic, and well-conserved degradative process which involves several molecular players and steps. Starting from the elongation of the phagophore, which leads to the autophagosome formation, the cytoplasmic material is eventually engulfed and degraded in the autophagolysosome after fusion with the lysosome (11). New building blocks and metabolites are now released and available for the cell. **(B)** Melanomagenesis from melanocytic nevi is blocked upon melanocytic-specific depletion of autophagy genes, such as *Atg5* and *Atg7* in GEMMs (64, 65). Syngeneic models displayed similar evidence, as in the case of host mice injected with *Beclin1*-engineered B16-F10 melanoma cells (67) or upon injection of melanoma cells in *Atg7*- and *Parkin*- genetically engineered mice (68, 69). **(C)** Melanoma development is favored upon single-copy loss of *Atg5* in GEMM (65). Primary tumors from melanoma patients have been found to have reduced expression of ATG5, if compared to melanocytic nevi (17). Therefore, impaired autophagy, as well as putative additional functions of Atg5 can induce melanoma.

Macroautophagy (hereafter referred to as autophagy) is a highly-conserved self-degradative process that allows the cell to deliver unwanted cargo, such as damaged proteins and organelles, to the lysosome for degradation (6–8). So-called “bulk” autophagy virtually occurs in all cells as a general homeostatic process. Notably, in response to certain molecular triggers, cells are also able to dispose of specific cargo by selective autophagy (e.g., mitophagy, the selective removal of damaged mitochondria) (8, 9). This selectivity is mostly determined by specific protein receptors, such as SQSTM1/p62 or OPTN (9). Regardless of cargo specificity, autophagic flux is tightly regulated by two major molecular sensors, mTORC1, which acts as a nutrient sensor, mostly for amino acids, (10), therefore negatively regulating autophagy, and AMPK, which detects shifts in the AMP/ATP ratio in the cell, consequently promoting autophagy (6). In order to restore balance in cell energy supply and demand, AMPK, in an mTORC1-dependent or independent fashion, initiates an intricate signaling cascade (6). All these signals converge to the ATG proteins, which assemble in four molecular complexes to form the core autophagic machinery: the ULK1 kinase complex (ULK1, ATG13, ATG101, and FIP200), the PI3KIII complex (VPS34, VPS15, ATG14, and BECLIN1), the ubiquitin-like conjugation system (ATG3, ATG4, ATG5, ATG7, ATG10, ATG12, ATG16, and LC3/GABARAP) and transmembrane protein like ATG9 (11). Altogether, these complexes promote the initiation, elongation, formation, and maturation of autophagosomes (Figure 1A) (11, 12).

In the aim of discovering novel anti-tumor therapies, autophagy has, over the past years, been investigated with great interest as a process that could potentially be modulated in tumor cells for the benefit of cancer patients (13). In melanoma, autophagy seems to play a complex and dynamic role which highly depends on the progression stage of the disease, the metabolic demand of the tumor as well as intrinsic (tissue microenvironment -TME, immunity) and extrinsic aspects (therapies) of the disease (6, 7, 14). To address this level of complexity in a clinically relevant system, syngeneic and genetically engineered mouse models (GEMMs) have been developed to fully recreate tumor progression from initiation to invasion and metastasis and to better characterize tumor-host interactions (15, 16). In this review, we will discuss how the different roles of autophagy can contribute to melanoma initiation and progression and delineate the precious insights that GEMMs and syngeneic mouse models have been able to provide to this field.

## Autophagy During Melanoma Evolution: A Tumor Suppressive Role?

The first studies aiming at understanding the contribution of autophagy to melanomagenesis and melanoma development revealed that, when impaired in melanocytes, autophagy can promote *BRAF*^*V*600*E*^-driven tumorigenesis, thus pointing to a tumor suppressor function for autophagy (17, 18). Importantly, autophagy seems to support BRAF^V600E^-dependent oncogene-induced senescence in melanocytes (17, 18). In particular, Liu et al. have found that a key autophagy gene (*ATG5*) is frequently down-regulated in primary melanomas when compared to benign nevi, strongly correlating with reduced progression-free survival in a cohort of early stage cutaneous melanoma patients (17). Other works have also shown that the expression of autophagy markers, such as LC3 and BECLIN1, was altered in melanocytic neoplasms, in a way that resulted in severely blunted autophagy during the early stages of melanocyte malignant transformation (19–22). This tumor suppressor role of autophagy at the onset of melanoma, as for other cancer types, has often been explained by the common view that autophagy-mediated removal of damaged organelles and redox-active protein aggregates might prevent the accumulation of detrimental and dysfunctional material, eventually preserving bioenergetics and redox metabolism (23, 24). Indeed, loss of autophagy has been often associated with increased oxidative stress, mostly as a consequence of accumulation of damaged mitochondria (see below), genomic instability and alteration of cell growth pathways, all circumstances leading to malignant transformation (23, 25). However, the mechanistic studies unraveling the real cause-effect relation between autophagy loss and melanoma initiation are still incomplete and in need of further investigation. Interestingly, Li and collaborators have recently shown that autophagy is restrained in melanoma cells at a transcriptional level in a BRAF^V600E^-dependent fashion in a syngeneic mouse model of melanoma (26, 27). Briefly, they have found that the master regulator of the expression of autophagy/lysosomal genes, TFEB, is under the control of the BRAF-oncogenic pathway, via its ERK-mediated phosphorylation and inactivation. This has been associated with TGFβ-signaling activation and induction of a metastatic phenotype that can be rescued by autophagy activation through the block of TFEB ERK-mediated phosphorylation and activation. This phenomenon was reverted (back to the metastatic phenotype) upon BRAF inhibition (26). Of note, BRAF^V600E^ activity has also been shown to inhibit AMPK by compromising its interaction with LKB1, thereby promoting melanoma cells proliferation (28). Therefore, the oncogenic pathway orchestrated by BRAF^V600E^ seems to be tightly involved in repressing autophagy, supporting the initial concept that loss of autophagy promotes melanomagenesis. The importance of understanding the intricate crosstalk between autophagy (and lysosomal-associated functions) and the oncogenic pathways activated during the onset of melanoma is supported by the evidence that melanoma cells highly depend on lysosome-associated vesicular trafficking in the very early stages of melanoma development (29). Moreover, it is noteworthy that, although the master regulators of melanocyte development MiTF, TFEB, and TFE3 belong to the same MiT/TFE transcription factor family (30), their activity correlates with the expression of diverse lysosomal and autophagy genes (31). Indeed, while it is known that MAPK/ERK signaling is involved in MiTF turnover and activation, with a crucial contribution of BRAF-mediated transcriptional and post-transcriptional regulation (32, 33), the recently discovered ERK-mediated signaling pathway that controls TFEB in *BRAF*^*V*600*E*^-driven melanoma does not work in the same way for either MiTF or TFE3 (26). To further complicate this scenario, the MiT/TFE transcription factor family has been also reported to finely regulate the lysosomal activity of mTORC1, the master regulator of cell growth as well as autophagy inhibitor (10), through direct regulation of RagD GTPase expression (34). Di Malta and colleagues elegantly demonstrated that the growth of melanoma, as for other cancer types, is deeply affected by the activity of MiT/TFE transcription factors which, besides regulating autophagy, lysosomal, and melanosome biogenesis, are specifically deputed to positively control lysosomal recruitment of mTORC1, thus enabling its activation and promoting cancer growth (34). In relation to mTORC1 activation, it is also worth mentioning that Bosenberg's group thoroughly demonstrated that mTORC1 activation, through loss of the Lkb1/AMPK pathway, is not sufficient to induce melanomagenesis in a *Braf*^*V*600*E*^-driven melanoma model (35). Indeed, melanoma development requires the concomitant activation of mTORC1 and 2 in a GEMM carrying the *Braf*^*V*600*E*^ mutation uniquely in melanocytes (35). However, the link to autophagy function has not been unraveled yet in this specific context. Indeed, though providing possible clues, all these discoveries still puzzle the intricate scenario of the signaling cascades activated to control autophagy during melanomagenesis.

That said, a growing body of evidence has been pointing out a controversial *oncogenic* function to autophagy during melanomagenesis. Herein, we will dissect the possible explanations of such a contradictory view and how the application of GEMMs and syngeneic models (15, 16) have emerged to elucidate this complex function of autophagy in melanoma.

## Autophagy in Melanoma Biology: An Oncogenic Role?

It is worth underlining that autophagy is principally meant as a key survival mechanism for the cell. Indeed, autophagy enables cells to recycle building blocks and metabolic substrates (primarily carbohydrates, fatty acids -FAs, amino acids, and nucleosides/nucleotides) needed for continuous growth and for sustaining the adaptive high metabolic demand cells require upon diverse stress conditions (23). This places autophagy at a crossroad with cell metabolic rewiring, a strategy adopted by melanoma cells to sustain a constant growth and metastatic progression (36). In this section, we will sum up the latest findings emphasizing the essential role of autophagy in supporting melanoma growth and metastasis, pointing out autophagy as an oncogenic/metabolic machinery in melanoma.

### Metabolic Pathways in Melanoma

Metabolic reprogramming is considered one of the hallmarks of cancer, being involved in cancer initiation, maintenance, and progression (37). Historically, glycolysis represents the central metabolic pathway implicated in melanoma evolution, with the Warburg effect, i.e., the preferential use of aerobic glycolysis to oxidative phosphorylation (OxPHOS) for ATP production, having a predominant role (38–41). The glycolytic pathway of melanoma cells intrinsically relies on the activation of signaling pathways, such as (i) the MAPK pathway that, hyperactivated in BRAF^V600E^-driven melanomas, controls HIF-1α and MYC activities (42, 43) and (ii) the PI3K/AKT/mTOR pathway which is hyperactivated upon loss of PTEN [another common alteration found in melanoma patients (44)] or upon AKT and PI3K activating mutations (45, 46). Importantly, mitochondrial OxPHOS can also be hyperactivated in melanoma cells, mainly through the master regulator of energy metabolism PGC-1α, which was found highly expressed in a subset of melanomas (39, 41, 47, 48).

While metabolic rewiring allows cancer cells to find alternative sources of energy to adapt to nutrient and oxygen limitation, tumor cells may also control nutrient demand indirectly by modulating protein synthesis. This strategy, extensively reviewed by García-Jiménez and Goding, is present in many cancer types including melanoma (49). Indeed, recent findings suggest that translation reprogramming in melanoma can be initiated by different stress kinases and is mainly orchestrated via phosphorylation of the eukaryotic translation initiation factor eIF2α (50–52). This reprogramming can be triggered by various molecular cues such as oncogenic BRAF-induced ER stress (53–55), amino acid limitation (50, 51) or inflammation (51), that ultimately converge toward reducing global protein translation and inducing autophagy (49, 50, 53, 54). In this context, the downstream autophagic response seems to be an essential pro-survival mechanism that not only allows melanoma cells to thrive but also promotes an invasive phenotype (50, 52, 55). Of note, ER stress induced autophagy can also occur in an eIF2α-independent fashion via JNK signaling (53, 54).

Recently, a key role has emerged also for lipid metabolism in melanoma. As lipids are a source for membranes and a reservoir for ATP and acetyl-CoA, it is not surprising that FAs synthesis and oxidation have been entangled in melanoma growth and progression (39, 56). Interestingly, Zhang and colleagues have recently demonstrated that fatty acid transporters, such as FATP1, are highly expressed in melanoma (56). In this way, melanoma cells metabolism was considerably affected and melanoma progression sustained by increased uptake of FAs from TME-resident adipocytes, hence revisiting FATPs as new possible targets in melanoma therapy (56). Moreover, beyond cell energetic requirements, lipid alterations can also affect lipid signaling in melanoma (57, 58). Indeed, FAs can bind and activate specific nuclear receptors, i.e., PPARs, thus controlling the expression of genes involved in lipid homeostasis, oxidative stress, and inflammation (59, 60). Unfortunately, poor knowledge exists on this matter as the only evidence for a role of lipid signaling in melanoma biology comes from two recent papers in which the authors independently remarked an anti-tumor effect of PPARβ/δ and PPARγ in melanoma progression and metastasis (61, 62).

### Autophagy as Metabolic Pathway: Cell-Autonomous Autophagy

By making available different sources of energy and by directly controlling mitochondria homeostasis through selective removal of mitochondria (see below), autophagy is unquestionably a central player in tumor metabolism (36). Indeed, recent evidence demonstrated a tight interplay between autophagy and lipid metabolism, proposing autophagy as a mechanism regulating both β-oxidation and production of ketone bodies (63).

The most emerging discoveries posing the question on autophagy as promoting or suppressive mechanism during melanoma evolution come from studies performed in GEMMs (Table 1). Results from a very sophisticated *Braf*^*V*600*E*^-mutation and *Pten*-deletion tamoxifen-inducible mouse model of melanoma, which develops cutaneous melanoma resembling the human disease (70, 71), have mainly flagged an oncogenic role for autophagy in melanoma development, highlighting the complexity of this process in the malignant transformation of melanocytes. In particular, melanocyte-specific *Atg7* or *Atg5* ablation can prevent melanoma formation and drive melanocytic senescence in the same GEMM model (Figure 1B) (64, 65), being the *Atg7*-related phenotype associated with increased oxidative stress and extended survival rate (64). In this context, autophagy clearly seems to harbor an oncogenic role. Strikingly, hemizygous deletion of *Atg5* enhances melanoma growth (compared to *Atg5*^+/+^ or *Atg5*^−/−^), metastatic power and resistance to targeted therapy, such as BRAF inhibitor (Figure 1C) (65). Of note, as for the *Atg7*-deficient *Braf*^*V*600*E*^; *Pten*^−/−^ driven melanomas (64), complete loss of *Atg5* reduced tumor formation (65). Importantly, single-copy loss of *ATG5* has been identified as a distinctive feature of advanced melanomas, regardless of the mutational status of melanoma associated-oncogenes (e.g., *BRAF, RAS*), and associated with poor overall patient survival (Figure 1C) (65). These very interesting findings add another layer of complexity to the general knowledge about the impact of autophagy on melanoma evolution, even arguing for a dose-dependent contribution of autophagy genes, as seen for *Atg5*, and opening new hypotheses on additional autophagy-unrelated function(s) of Atg5 (72). In line with this idea, a very recent work indicates that the well-known autophagy receptor Sqstm1/p62 is involved in fueling melanoma progression through a distinct non-autophagy pathway (66). In particular, the authors demonstrated that p62 can positively regulate the mRNA stability of a cluster of pro-tumorigenic factors in melanomas, thus arguing for p62, an alternative oncogenic role which seems to be far from its autophagy function, at least in this type of tumor (66).

**Table 1:** Melanoma mouse models.

	***In vivo* model**	**Target gene**	**Cellular function**	**Target pathway**	**Outcome**	**References**
*Genetically modified models*	*Tyr::Cre^*ERT*2/+^* *LSL-Braf^*V*600*E*/+^* *Pten^*FLOX*/*FLOX*^* *Atg7 ^*FLOX*/*FLOX*^*	*Atg7*	Autophagosome formation	Autophagy	Reduced melanoma growth	(64)
	*Tyr::Cre^*ERT*2/+^* *LSL-Braf^*V*600*E*/+^* *Pten^*FLOX*/*FLOX*^* *Atg5 ^*FLOX*/+^*	*Atg5*	Autophagosome formation	Autophagy (and ?)	Increased melanoma growth and metastasis	(65)
	*Tyr::Cre^*ERT*2/+^* *LSL-Braf^*V*600*E*/+^* *Pten^*FLOX*/*FLOX*^* *Atg5 ^*FLOX*/*FLOX*^*	*Atg5*	Autophagosome formation	Autophagy	Reduced melanoma growth	(65)
	*Tyr::Cre^*ERT*2/+^* *LSL-Braf^*V*600*E*/+^* *Cdkn2A^*FLOX*/*FLOX*^* *Lkb1^*FLOX*/*FLOX*^*	*Lkb1*	Control of AMPK and AKT pathways	mTORC1 and mTORC2 mediated signaling	Development and progression of melanoma	(35)
	*Tyr::Cre^*ERT*2/+^* *LSL-Braf^*V*600*E*/+^* *Pten^*FLOX*/*FLOX*^* *Sqstm1/p62^-/-^*	*Sqstm1/p62*	Autophagy, protein ubiquitination	mRNA decay	Reduced melanoma growth and metastasis to LNs	(66)
*Syngeneic melanoma models*	HMM: C57Bl/6 MCL: *Beclin1^-/-^* B16-F10	*Beclin1*	PI3K complex activation and autophagy induction	Autophagy	Reduced melanoma growth	(67)
	HMM: C57Bl/6J *Atg7^-/-^* MICL: *Braf^*V*600*E*/+^, Pten^-/-^*, *Cdkn2a^-/-^*	*Atg7*	Autophagosome formation	Autophagy	Reduced melanoma growth	(68)
	HMM: C57Bl/6J MCL: *Braf^*V*600*E*^, TFEB^*S*142*A*^* or *TFEB^*S*142*E*^* B16-F10	*TFEB*	Transcription factor involved in autophagy/lysosomal genes regulation	Autophagy	Reduced (TFEB^S142A^) or increased (TFEB^S142E^) melanoma metastasis	(26)
	HMM: C57Bl/6 *Parkin^**-/-**^* MCL: B16-F10	*Parkin*	Mitophagy, protein ubiquitination	Mitophagy	Reduced melanoma growth and metastases	(69)

*Two main types of mouse models are currently used for melanoma research: genetically modified mouse models (GEMMs) or syngeneic melanoma models. GEMMs harbor melanoma-driving genomic alterations, such as loss of either Pten (Pten^FLOX/FLOX^) or Cdkn2a (Cdkn2a^FLOX/FLOX^) in combination with single-allele mutation of Braf (Braf^V600E/+^) (35, 70, 71). The expression of these mutations is finely regulated as achievable exclusively by activation of an inducible melanocyte-specific recombinase system (Tyr::Cre^ERT2^). The effect(s) of a specific target gene (e.g., an autophagy gene) on melanoma biology can be accomplished by introducing a Cre-mediated (complete or not) deletion of a gene of interest. The use of GEMMs is of relevant interest for studying melanoma initiation, development, progression and anti-cancer therapy. Syngeneic mouse models consist of implantation or injection of tumor tissues or genetically (or not) modified murine cell lines in a host mouse, which can also be genetically modified for a gene of interest. The use of syngeneic models is of relevant interest for studying melanoma biology and host-tumor biology, especially in the context of immunotherapy as the host retains an intact immune system. HMM, host mouse model; MCL, melanoma cell line; MICL, melanoma isogenic cell line*.

### Mitochondria in Melanoma

Considering the intricate role played by mitochondria in maintaining metabolism and cellular homeostasis, it is not surprising that alterations in mitochondria may occur and be involved in melanoma features. Indeed, disruption of mitochondrial capacity has been implicated in reduced melanoma cell growth in an *in vivo* mouse xenograft model (73).

Even though mitochondrial bioenergetics largely sustains both the high proliferation rate and the energetic demand of cancer cells (74), mitochondrial metabolism can also act as a double-edged sword as mitochondria are primary producers and targets of reactive oxygen species (ROS). Although enhanced ROS production can activate signaling cascades that are favorable for tumorigenesis (75), the role of ROS in melanoma biology is still controversial and widely debated. A recent study revealed that scavenging ROS ensures maintenance of melanoma cells proliferation and migration both *in vitro* and in an *in vivo* xenograft tumor growth model (76). Bagati and colleagues shed further light on such a complex role by suggesting a double and stage-specific role for ROS in melanoma. Indeed, while mechanistically demonstrating that ablation of *Klf9*, a pro-oxidant transcriptional regulator, promotes metastases in a *Braf*^*V*600*E*^; *Pten*^−/−^ GEMM of melanoma without affecting primary tumor growth, the authors also reasoned that *Klf9* deficiency inhibits premalignant melanocytic hyperplasia in a *Braf*^*V*600*E*^-induced hyperplasia model (77).

On the other hand, massive mitochondrial oxidative damage can occur upon excessive ROS production and culminate in the activation of mitophagy, the autophagy-mediated mechanism that, tightly coordinating with the mitochondrial fission/fusion machinery, controls the clearance of dysfunctional mitochondria (78). As such, mitophagy can be exploited by cancer cells to isolate and degrade damaged mitochondria to ensure qualitatively functional organelles. Indeed, a reduced proliferative rate has been associated with compromised fission machinery and retention of dysfunctional mitochondria in melanoma cell lines (69, 73). Also, increased hyper-activation of the fission machinery, which has been positively correlated to the *BRAF*^*V*600*E*^ mutation in human patients, has been implicated in high proliferation of melanoma cells (79).

Unfortunately, little is known about the role of mitophagy regulators in melanoma biology. However, some evidence speaks in favor of an oncogenic role of these proteins in melanoma. As a matter of fact, the activity of BNIP3 has been discovered to crucially regulate removal of ROS-producing mitochondria. Doing so, BNIP3 favors mitochondrial fitness and maintains survival, bioenergetics, growth and aggressiveness of melanoma cells (80, 81). In parallel, Lee et al. have recently used an elaborate syngeneic model of B16-F10 melanoma cells injected in a transgenic *Parkin* knock-out mouse and assessed that Parkin favors melanoma growth and distal metastases by preventing the activation of the apoptotic cascade (Figure 1B) (69).

### Autophagy as Metabolic Pathway: Non-cell-Autonomous Autophagy

Over the last years, several researchers have attempted to address the interesting contribution of TME and systemic non-cell autonomous autophagy to tumor growth. Here, we report the most relevant publications on this matter that are using GEMMs or other genetically modified organism-based studies (Table 1).

White's group has recently demonstrated that, once autophagy is systemically depleted in a mouse model with conditional whole-body *Atg7* deficiency or when autophagy-proficient melanoma cells are subcutaneously injected into mice, melanoma growth significantly slows down (Figure 1B) (68). The authors neatly proved that, in the context of arginine auxotrophic melanoma (82), host autophagy is necessary to sustain tumor growth by systemically replenishing arginine. According to the authors, autophagy-deficient hosts release hepatic ARG1 to process circulating arginine. Even though the mechanism still needs to be elucidated, this interplay is essential for tumor growth (68). This important discovery further underpins the relevance of autophagy in supporting tumor growth: though cancer cells are autophagy-proficient, maintenance of their growth requires an autophagy-competent environment. Moreover, the whole scenario suggests that also restricting the availability of essential tumor nutrients deserves to be exploited as a strategy for improving melanoma therapy.

Of note, this discovery has shed light on the non-cell-autonomous contribution of autophagy to tumor growth, which was already supported by the elegant work of Katheder and colleagues that demonstrated the extremely relevant role of TME autophagy in promoting tumor development (83). Briefly, they generated sophisticated transgenic models of *Drosophila* where different autophagy depletion assets were applied, in order to distinguish the contribution of tumor-only, TME-only (or the combination of both), and of host autophagy to tumor growth. Their interesting results displayed a more significant reduction of tumor growth and invasion in the neighboring tissue when local- and distal-autophagy deficiency were induced, giving enormous relevance to the microenvironmental signaling component (e.g., chemotactic cytokines) and to the metabolic addiction of the tumor to the microenvironment (83).

## Conclusions and Perspectives

The last breakthroughs in dissecting the role of autophagy in melanomagenesis and melanoma progression have brought the metabolic aspects of autophagy to our attention, revealing autophagy as a fundamental machinery that sustains tumor metabolism. This implies the autophagic process to be pro-tumorigenic in the evolution of melanoma, especially supporting tumor growth (Figure 1B). It becomes even more relevant when considering that autophagy activation in both tumor cells and host can all affect melanoma development. The application of genetically engineered or syngeneic melanoma mouse models improved our understanding of these phenomena (Figure 1) and, most importantly, allowed researchers to test the therapeutic advantages of blocking autophagy in the treatment of melanoma, the beneficial effects of which have already been emphasized (13, 84, 85).

One issue, that has yet to be mentioned, is the contribution of autophagy to the regulation of the antitumor immunity and how this can influence the development of melanoma. As many other cancer types, melanoma can provoke an intricate and multifaceted immune response which can be more or less deleterious for the tumor, depending on several factors (86, 87). The involvement of autophagy in this context can very much depend on where it is exerting a major function in regulating the immune response and which immune cells are sensing and then responding to the different levels of autophagy (88–91). For instance, the group of Verginis demonstrated that autophagy inhibition in myeloid-derived suppressor cells (MDSCs), a population of immune cells accumulating within the tumors with the function of suppressing the immune response (92), can redirect these cells toward an antitumor immune reaction (93). In particular, they observed that MDSCs in both melanoma patients and mouse models displayed high autophagy levels. Therefore, by interfering with the autophagy status specifically in MDSCs, they successfully re-activated the tumor-specific CD4 positive T-cells and repressed the MDSCs suppressive function toward the tumor, with a resulting remarkable delay in tumor growth (93). Another interesting example is the study of Mgrditchian and co-workers, where the authors thoroughly demonstrated that inhibition of autophagy in melanoma cells negatively affected tumor growth by favoring the infiltration of natural killer (NK) cells into the tumor (67, 94–96). Placing autophagy again as an oncogenic process, Mgrditchian et al. demonstrated that syngeneic melanoma models depleted of *Beclin1* or other autophagy genes (*Atg5, Sqtsm1/p62*) or with pharmacologically-inhibited autophagy (e.g., with chloroquine), showed significantly decreased melanoma development due to an enhanced NK cells-mediated immune response (Figure 1B) (67, 94). Taken together, these findings provide further insights into the oncogenic role of autophagy and how it can influence tumor growth by modulating the immune response, a highly relevant matter considering that melanoma is a great candidate for immunotherapy application (3, 5). This other layer of regulation by autophagy therefore undoubtedly bears a high clinical relevance for the future and could open new venues for autophagy-targeted therapy in melanoma.

## Author Contributions

DD, LD, and VB wrote the manuscript. LD and DD drafted the figure and table. DD provided senior supervision and critically revised the manuscript.

### Conflict of Interest

The authors declare that the research was conducted in the absence of any commercial or financial relationships that could be construed as a potential conflict of interest.

## Abbreviations

AKT: AKT serine/threonine kinase 1
AMPK: 5′ adenosine monophosphate-activated protein kinase
ARG1: arginase 1
ATG: autophagy-related gene
BNIP3: BCL2-interacting protein 3
BRAF: v-raf murine sarcoma viral oncogene homolog
CD4: cluster of differentiation 4
CTLA-4: cytotoxic T-lymphocyte-associated protein 4
eIF2α: eukaryotic initiation factor 2 alpha
ER: endoplasmic reticulum
ERK: extracellular signal-regulated kinase
FA: fatty acid
FATP1: fatty acid transfer protein 1
FIP200: focal adhesion kinase-interacting protein 200 kDa
GABARAP: GABA type A receptor-associated protein
GEMM: genetically engineered mouse model
HIF-1α: hypoxia inducing factor 1 alpha
JNK: jun N-terminal kinase
Klf9: Krueppel-like factor 9
LC3: light chain 3
LKB1: liver kinase B1
MAPK: mitogen-activated protein kinase
MDSCs: myeloid-derived suppressor cells
MEK: MAPK/ERK kinase 1
MiTF: melanocyte inducing transcription factor
mTORC1: mammalian target of rapamycin complex 1
MYC: myelocytomatosis oncogene cellular homolog
NK: natural killer
NRAS: neuroblastoma RAS viral oncogene homolog
OPTN: optineurin
OxPHOS: oxidative phosphorylation
PD-1: programmed cell death protein 1
PD-L1: programmed cell death ligand 1
PGC-1α: peroxisome proliferator-activated receptor-gamma coactivator 1 alpha
PI3K: phosphatidylinositol-3-kinase
PI3KIII: class III phosphatidylinositol-3-kinase
PPAR: peroxisome proliferator-activated receptor
PTEN: phosphatase and tensin homolog
RagD: Ras-related GTP-binding protein D
RAS: rat sarcoma
ROS: reactive oxygen species
SQSTM1: sequestosome 1
TFEB: transcription factor EB
TFE3: Transcription Factor Binding To IGHM Enhancer 3
TGF-β: tumor growth factor beta
TME: tissue microenvironment
ULK1: unc-51 like autophagy activating kinase 1
Vps 15 34: vacuolar protein sorting 15 34.
